# CXCR4/CXCL12 expression and signalling in kidney cancer

**DOI:** 10.1038/sj.bjc.6600221

**Published:** 2002-04-22

**Authors:** A J Schrader, O Lechner, M Templin, K E J Dittmar, S Machtens, M Mengel, M Probst-Kepper, A Franzke, T Wollensak, P Gatzlaff, J Atzpodien, J Buer, J Lauber

**Affiliations:** Department of Cell Biology and Immunology, German Research Centre for Biotechnology (GBF), D-38124 Braunschweig, Germany; Department of Visceral Surgery and Urology, Klinikum Braunschweig, D-38126 Braunschweig, Germany; Department of Urology, Medizinische Hochschule Hannover (MHH), D-30623 Hannover, Germany; Department of Pathology, Medizinische Hochschule Hannover (MHH), D-30623 Hannover, Germany; Department of Haematology and Oncology, Medizinische Hochschule Hannover (MHH), D-30623 Hannover, Germany; Institut of Medical Microbiology, Medizinische Hochschule Hannover (MHH), D-30623 Hannover, Germany

**Keywords:** CXCR4, CXCL12, kidney cancer, receptor signalling, functional genomics

## Abstract

CXCL12 (SDF-1), a CXC-chemokine, and its specific receptor, CXCR4, have recently been shown to be involved in tumourgenesis, proliferation and angiogenesis. Therefore, we analysed CXCL12α/CXCR4 expression and function in four human kidney cancer cell lines (A-498, CAKI-1, CAKI-2, HA-7), 10 freshly harvested human tumour samples and corresponding normal kidney tissue. While none of the analysed tumour cell lines expressed CXCL12α, A-498 cells were found to express CXCR4. More importantly, real-time RT–PCR analysis of 10 tumour samples and respective adjacent normal kidney tissue disclosed a distinct and divergent downregulation of CXCL12α and upregulation of CXCR4 in primary tumour tissue. To prove that the CXCR4 protein is functionally active, rhCXCL12α was investigated for its ability to induce changes of intracellular calcium levels in A-498 cells. Moreover, we used cDNA expression arrays to evaluate the biological influence of CXCL12α. Comparing gene expression profiles in rhCXCL12α stimulated *vs* unstimulated A-498 kidney cancer cells revealed specific regulation of 31 out of 1176 genes tested on a selected human cancer array, with a prominent stimulation of genes involved in cell-cycle regulation and apoptosis. The genetic changes reported here should provide new insights into the developmental paths leading to tumour progression and may also aid the design of new approaches to therapeutic intervention.

*British Journal of Cancer* (2002) **86**, 1250–1256. DOI: 10.1038/sj/bjc/6600221 www.bjcancer.com

© 2002 Cancer Research UK

## 

Chemokines constitute a superfamily of small pro-inflammatory cytokines that are involved in a variety of immune reactions including allergy, inflammation, infection, tissue injury, cardiovascular diseases and malignant tissue growth (Wang *et al*, 1998). Especially in solid tumours, recent research focused on the subfamily of α- or CXC-chemokines.

The CXCL12 (=SDF-1) gene has been localised to chromosome 10q11.1, which is in contrast to most genes of the other known CXC chemokines that cluster on chromosomes 4. The ubiquitous expression of CXCL12 is consistent with the presence of TATA-less and GC-rich sequences in the 5′-flanking region, as is often found in so-called ‘housekeeping genes’ (Shirozu *et al*, 1995). Recent studies indicate that CXCL12 expression is markedly decreased in rapidly dividing fibroblasts and liver cells (Jiang *et al*, 1994), premalignant colonic adenomas, hepatocellular carcinomas (Shibuta *et al*, 1997), as well as in various malignant cell lines (Begum *et al*, 1996, 1999). Unlike CXCL12, CXCR4 expression has been shown to be heterogeneous in malignant cells of different origin. While it was reported to be markedly decreased in hepatocellular carcinomas (Begum *et al*, 1999) and increased in glioblastoma multiforme, breast and uterine cancer as well as Burkitt's lymphoma (Sehgal *et al*, 1998a; Rempel *et al*, 2000), it was not found to be regulated in colon, oesophageal and gastric cancer (Mitra *et al*, 1999), or in different tumour cell lines (Dwinell *et al*, 1999; Murdoch *et al*, 1999).

In this study we have looked for chemokine receptor expression in four primary human kidney cancer cell lines, 10 freshly harvested human tumour samples and the corresponding normal kidney tissues. Furthermore, we have identified key molecular changes induced upon CXCL12/CXCR4 signalling by means of a cDNA array approach. Genetic changes reported here may provide new insights into the developmental paths leading to tumour progression and may also aid the design of new approaches to therapeutic intervention.

## MATERIALS AND METHODS

### Patients

Written informed consent was obtained from all patients before entry into the study. Renal cell carcinoma (RCC) and adjacent normal kidney tissue samples were collected from 10 patients immediately after tumour nephrectomy. All patients presented with RCC of the clear cell type and were treated at the Staedtischen Klinikum Braunschweig between January and June 2001. As patient 6 presented with a large tumour, which macroscopically appeared to consist of two areas of different cell types, two samples were taken thereof. Patient 10 suffered from a small tumour that had not increased in size for more than 12 months before surgery and macroscopically appeared like a benign tumour. Patient characteristics are summarised in Table 1

**Table 1:**
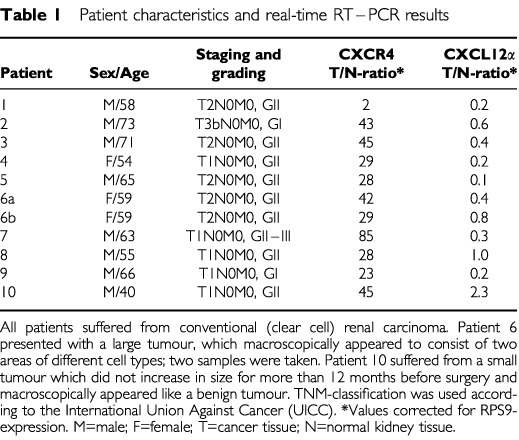
Patient characteristics and real-time RT–PCR results

.

### Cell lines

Human kidney cancer cell lines A-498, CAKI-1, CAKI-2 and HA-7 (DSMZ Braunschweig, Germany) were grown in RPMI medium supplemented with 10% heat-inactivated foetal bovine serum (FBS; Bio Whittaker Europe, Belgium), 2 mM L-glutamine (Gibco BRL, Scotland, UK), 10 U ml^−1^ penicillin and 10 mg ml^−1^ streptomycin (Sigma, Germany); cells were maintained in 95% air/5% CO_2_ at 37°C.

### RT–PCR

Tumour and kidney tissue samples were processed immediately after surgery. Total RNA was extracted using TriReagent (MRC-Research, USA), and reverse transcribed with M-MLV reverse transcriptase (Gibco, Scotland, UK) and Oligo dT-primers (Gibco, Scotland, UK) for 35 min at 42°C, according to the manufacturers instructions. The reaction was stopped by the addition of 40 μl Tris-EDTA(TE)-buffer (pH=8) and heat-inactivation for 8 min at 90°C. The following primers (MWG-Biotech, Ebersberg, Germany) were used for the subsequent PCR: CXCR1 (sense: 5′ CCT TGG GGG TGG TGG AT 3′; antisense: 5′ AGG GCT GCT TGT CTC GTT C 3′), CXCR2 (sense: 5′ TGC CTC ACC CCT TGC CAT AA 3′; antisense: 5′ GCC AGC CTG ATT TTC TTT CTT TTG 3′), CXCR3 (sense: 5′ GTG GCC GAG AAA GCA GGG TAG ACG 3′; antisense: 5′ CAG GCG CAA GAG CAG CAT CCA CAT 3′), CXCR4 (sense: 5′ GCC TTA TCC TGC CTG GTA TTG TC 3′; antisense: 5′ GCG AAG AAA GCC AGG ATG AGG AT 3′), CXCR5 (sense: 5′ CGG AAA GCA GCT CGA AGG CAC AGT 3′; antisense: 5′ CTC CGT TGG CAA GGG CAG AAG TAG 3′); CXCL12α (sense: 5′ ACT GGG TTT GTG ATT GCC TCT GAA 3′; antisense: 5′ GGA ACC TGA ACC CCT GCT GTG 3′). mRNA integrity and reverse transcription were checked with primers for the ribosomal protein S 9 (RPS9)-gene (sense: 5′ CGC AGG CGC AGA CGG TGG AAG C 3′; antisense: 5′ CGA AGG GTC TCC GCG GGG TCA CAT 3′), a common ‘housekeeping gene’. PCR was performed with Promega Taq polymerase (Promega, USA) following the manufacturers instruction. Cycling conditions: 5 min at 94°C, 30 cycles of 30 s at 94°C, 30 s at 54°C (CXCR1+2) or 58°C, 30 s at 72°C, and, finally, 10 min at 72°C. PCR products were resolved by electrophoresis on 1.5% agarose gels and visualised by ethidium bromide staining. Specificity of the amplified products was established by restriction enzyme digestion.

### Real-time RT–PCR

To quantify CXCR4-, CXCL12α- as well as RPS9-mRNA levels, we established a real-time RT–PCR assay. For this purpose, we used a GeneAmp® 5700 thermal cycler and the Taq-Man® Universal Master Mix (Perkin Elmer) including SYBR®-Green PCR Core Reagents, according to the manufacturer's instructions. Primers and cycling temperatures were identical with those used for conventional RT–PCR. All experiments were performed at least twice. A detailed description can be obtained upon request from the corresponding author.

### Flow cytometry

A-498 cells were removed from flasks non-enzymatically, washed, and resuspended at 10^6^ cells ml^−1^ in ice cold washing buffer (phosphate-buffered saline (PBS) containing 0.5% FBS) and incubated with 10 μg ml^−1^ R-phycoerythrin-labelled mouse anti-human CXCR4 antibody (12G5; PharMingen, San Diego, USA) for 90 min at 4°C. Cells were analysed with a FACScalibur flow cytometer (Becton Dickinson, USA).

### Calcium mobilisation assay

A-498 cells were grown on 8-chambered glass cover slips (Lab Tek Brand Products, USA) and loaded with 2 μM fura-2/AM (Molecular Probes, USA) for 45 min at 37°C. Cover slips were transferred into a FBS-free buffer and fura-2-specific fluorescence in individual cells was measured after addition of 2 μg ml^−1^ rhCXCL12α (R&D Systems, USA), using a confocal microscope (BIO-Rad, Germany) with an epifluorescence ×40 objective. Emission fluorescence at 510 nm was measured upon excitation at 340 and 380 nm. Data are presented by the ratio of fluorescence intensity at 340 nm divided by 380 nm. To establish the integrity of the epithelial cells, intracellular calcium was also measured using bradykinin (Sigma, Germany) as control stimulant.

### Immunohistochemistry

Formalin-fixed and paraffin-embedded archival specimens of clear cell renal carcinomas with adjacent normal kidney tissue were deparaffinised in xylol following standard methods. For antigen retrieval, heat pretreatment was performed using a microwave oven (citrate buffer, pH=6.2; 20 min, 600 W). After blocking endogenous peroxidase with 3% H_2_O_2_ and endogenous biotin by Avidin-Biotin-Blocking-Kit (Vector Laboratories, CA, USA) the polyclonal goat anti-human CXCR-4 (fusin, clone A-17; Santa Cruz Biotechnology, CA, USA) antiserum (1 : 500) was incubated overnight at 4°C. After washing thoroughly in TRIS buffered saline, a biotinylated secondary rabbit-anti-goat-antibody was incubated (1 : 250, 60 min). The biotin was detected by an optimised alkaline phosphatase conjugated streptavidin-biotin-complex (NEN Life Science, MA, USA). Fast red served as substrate and haemalaun for counterstaining.

### Molecular characterisation of CXCL12α/CXCR4 signalling

A-498 cells were grown in two 75 cm^2^ flasks to confluence. Subsequently, fresh culture medium was added to both flasks, in one flask combined with 0.5 μg ml^−1^ rhCXCL12α. Cells were incubated at 37°C for 6 h and 18 h, respectively, and immediately used for total RNA isolation. Differential expression of 1176 genes in rhCXCL12α stimulated *vs* unstimulated A-498 cells was investigated by cDNA array analysis using the Human Cancer 1.2 Array (Clontech, Germany). Sample preparation was performed as previously described (Eisen and Brown, 1999). Briefly, total RNA was extracted twice using TriReagent LS and a DNAse I digestion step in between. 3.5 μg of total RNA from stimulated and unstimulated cultures, respectively, were used as template for the specific synthesis of ^32^P-radiolabelled cDNA probes, according to the manufacturer's recommendations (Clontech, Germany), except that the RT-enzyme was replaced by Superscript II (Gibco, Scotland, UK). Subsequently, cDNA probes were hybridised side-by-side to identical gene array membranes (a complete list of cDNAs and controls immobilised on the Atlas Human Cancer 1.2 Array from Clontech can be found at http://www.clontech.com). After hybridisation and extensive washes, membranes were exposed to Phosphor Imaging Screens (Fuji, Japan). Radioactivity was detected using a Fuji Bas 2500 16-bit image analysis system. Spot density for each gene was measured using Array-Vision software (Version 5.1, Imaging Research, Canada), along with numerous quality control parameters (compare Array-Vision manual). Results were confirmed in two independent array analyses with consistent results. To compare density values between both arrays, the expression level of each gene was normalised to the total of all genes measured for each sample.

## RESULTS

### CXCR and CXCL12α mRNA expression

mRNA expression levels of chemokine receptors CXCR1-5 in the four renal cell cancer cell lines (A-498, CAKI-1, CAKI-2, and HA-7) were determined by RT–PCR. The only receptor to be consistently expressed in all, but especially in A-498 tumour cells was CXCR4 (data not shown).

**Figure 1:**
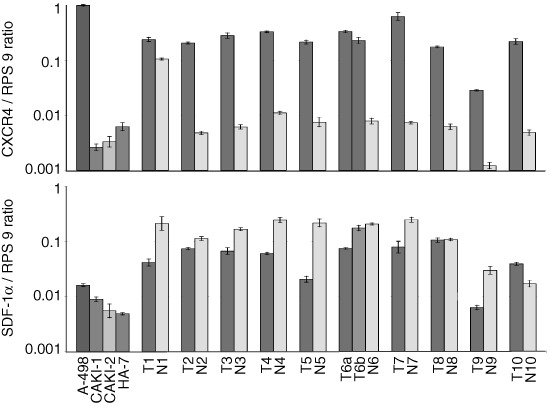
CXCR4 and CXCL12α mRNA expression using real-time RT–PCR. mRNA expression in four RCC cell lines (A-498, CAKI-1, CAKI-2 and HA-7), tumour (T) and adjacent normal non-malignant tissue (N) of 10 patients with RCC is demonstrated as a ratio corrected for that of RPS9 mRNA expression. The median T/N ratio for all patients was 29.6 (range, 2.3–85.2) for CXCR4, and 0.36 (range: 0.1–2.3) for CXCL12α, respectively, displaying a substantial upregulation of the receptor in malignant kidney tissue, while its ligand was less expressed compared to the corresponding normal renal tissue.

In order to quantify CXCR4 and CXCL12α expression levels, we used a real time RT–PCR assay. All four cell lines as well as 10 primary human tumour samples plus adjacent normal kidney tissues were tested for CXCR4 and CXCL12α as well as for RPS9-mRNA expression. The latter was used as independent ‘housekeeping-gene’. CXCR4 mRNA was particularly expressed in A-498 cells; CXCR4/RPS9 expression ratios for A-498, CAKI-1, CAKI-2, and HA-7 cell lines were 0.98, 0.002, 0.003 and 0.006, respectively (Figure 1

). On the other hand, CXCL12α was hardly expressed in any cell line (CXCL12α/RPS9 expression ratios ranging from 0.016–0.005; Figure 1), confirming the results gained by conventional RT–PCR.

In tumour and corresponding kidney tissue from the 10 patients with RCC, the distribution of tumour/kidney ratios for CXCR4 and CXCL12α gene expression, calculated by real time RT–PCR and corrected for that of RPS9 ((CXCR4/RPS9)_tumour_/(CXCR4/RPS9)_normal kidney_ and (CXCL12α/RPS9)_tumour_/(CXCL12α/RPS9)_normal kidney_, respectively), is given for each patient in Table 1 and Figure 1. The median tumour/kidney ratio was 29.6 (range, 2.3–85.2) for CXCR4, and 0.36 (range, 0.1–2.3) for CXCL12α, respectively, displaying a significant upregulation of CXCR4 in malignant kidney tissue, while its ligand CXCL12α was significantly lower expressed as compared to the corresponding normal renal tissue.

### CXCR4 surface expression

**Figure 2:**
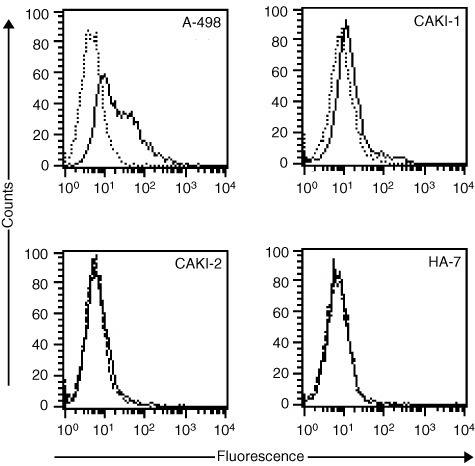
Flow-cytometric analysis of CXCR4 expression on A-498, CAKI-1, CAKI-2 and HA-7 RCC cell lines. The cell lines CAKI-2 and HA-7 did not express the receptor. In contrast, CAKI-1 showed weak and A-498 strong positive staining for the CXCR4 mAb 12G5.

CXCR4 cell-surface expression was evaluated by FACS analysis of all four RCC cell lines using a mouse anti-human CXCR4 and a matching IgG2a-isotype control antibody. Percentages of FACS-positive cells and relative mean fluorescence intensities (MFI) were determined. CAKI-2 and HA-7 cells turned out to be CXCR4 negative, while 7% of CAKI-1 cells (Δ relative MFI, 1.4) and 56% of A-498 cells (Δ relative MFI, 25.9) were CXCR4 positive, which was in well accordance with our RT–PCR results (Figure 2

).

**Figure 3:**
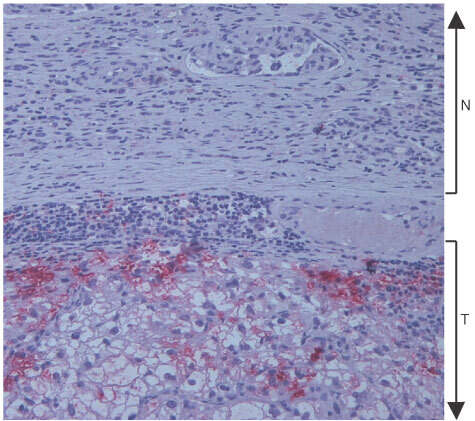
Immunohistochemical staining (biotin-streptavidin methodology) of a clear cell renal carcinoma of well to moderate differentiation with nearly diffuse cytoplasmatic expression of CXCR4 in malignant tissue (T) in contrast to adjacent normal kidney tissue (N).

Moreover, we used immunohistochemistry to confirm differential CXCR4 expression on protein level in clear cell kidney cancer. In contrast to the adjacent non-malignant kidney tissue, we were able to demonstrate a heterogeneous but constantly detectable expression of CXCR4 antigen in malignant tissue (Figure 3

).

### CXCR4 is functional

**Figure 4:**
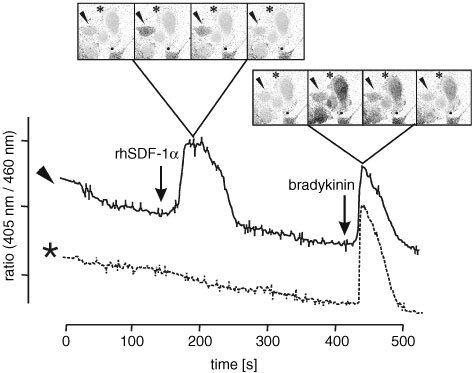
Ca^2+^ flux in A-498 cells in response to rhCXCL12α. Using confocal microscopy, we detected a heterogeneous reaction pattern of A-498 cells in response to rhCXCL12α stimuli. While stimulation with 2 μg ml^−1^ rhCXCL12α caused a rapid and robust increase of intracellular calcium in a fraction of A-498 cells (e.g. cell marked with an arrowhead), others did not respond to the stimulus (e.g. cell marked with a star). In contrast, a large increase in transient calcium was evoked by stimulation with 100 nmol bradykinin in all A-498 cells. Upper part of Figure=microscopy of single cells; lower part of Figure=intracellular Ca^2+^ flux visualised by fura-2 staining; arrow head, …rhCXCL12α responding A-498cell; *-rhCXCL12α non responding A-498 cell.

To determine whether the detected CXCR4 protein was functional, we analysed its ability to induce changes in intracellular calcium levels after rhCXCL12α stimulation. Simultaneous confocal microscopy-analysis of several cells revealed a heterogeneous reaction pattern of A-498 cells in response to rhCXCL12α stimulation. While 2 μg ml^−1^ rhCXCL12α caused a rapid and robust increase of intracellular calcium in a significant fraction of A-498 cells, others did not respond to the stimulus. In contrast, treatment with 100 nmol bradykinin induced a transient, large increase in calcium levels in all A-498 cells (Figure 4

).

### Molecular changes induced upon rhCXCL12α stimulation

In order to further analyse the molecular changes induced by CXCL12α/CXCR4 signalling, differential expression of 1176 genes in response to rhCXCL12α stimulation was investigated by cDNA array analysis. Therefore, A-498 kidney cancer cells were incubated either with or without rhCXCL12α for 6 h. Using background-threshold values of 1.4 and a minimal expression ratio of 1.9 (stimulated *vs* unstimulated), 31 genes were found to be significantly influenced by rhCXCL12α, which could be grouped into five major categories: genes involved in the CXCR4 signal transduction pathway, transcription factors, regulators of translation and the cell cycle, pro-apoptotic as well as miscellaneous genes such as PAI-1, interleukins, cathepsin L precursor, neuregulin and ECK (Table 2

**Table 2:**
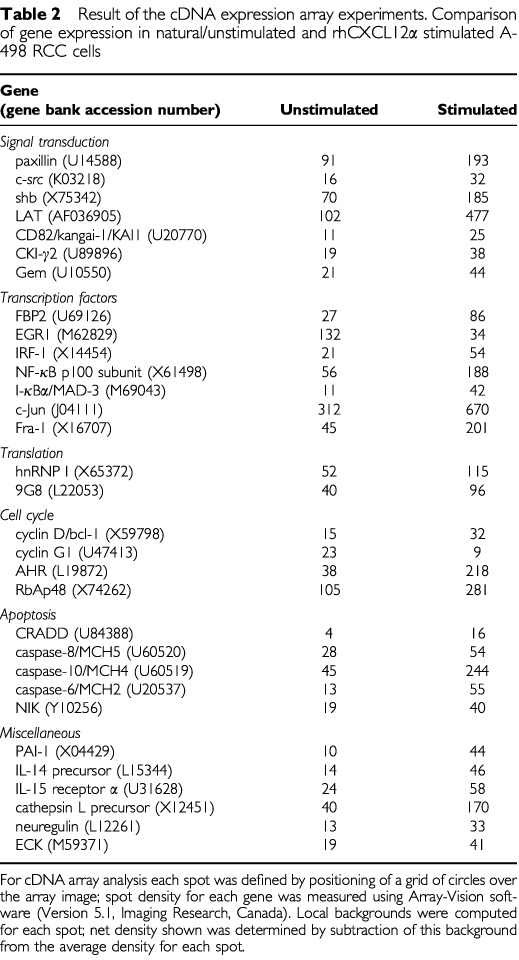
Result of the cDNA expression array experiments. Comparison of gene expression in natural/unstimulated and rhCXCL12α stimulated A-498 RCC cells

).

Moreover, we used a second array experiment to test the effects of rhCXCL12α on A-498 kidney cancer cells after 18 h of stimulation. Interestingly, CD82 was the only molecule that remained upregulated (factor, +2.2), while the transcription factor, c-*jun*, that was upregulated after 6 h were specifically downregulated after 18 h (factor, −1.9; data not shown).

## DISCUSSION

RCC, especially when metastasised, continues to be a frustrating tumour for clinicians to manage and treat. It is heterogeneous in appearance, displaying diverse histological as well as functional characteristics including cell proliferation, neoangiogenesis, necrosis and modulation of immune responses. Though most RCC exhibit a significant infiltration of host immune cells, mainly of the mononuclear lineage, the immune systems fails to establish adequate anti-tumour activity, inevitably leading to tumour progression and death.

Chemokines and their receptors are known to play an important role in the process of leukocyte trafficking and homing, especially at sites of inflammation, infection, tissue injury, cell damage and malignant tumour growth. Recent evidence suggests that these proteins can also regulate non-leukocyte cell functions such as angiogenesis/angiostasis, cell migration, activation of apoptotic cell death, and proliferation (Wang *et al*, 1998). Thus, we have investigated the expression of CXC-chemokine receptors on primary RCC cultures and on kidney cancer compared to normal kidney tissue, detecting an alteration of the CXCL12/CXCR4 signalling pathway in kidney cancer.

CXCR4 is one of the few chemokine receptors for which only a single ligand has been identified so far, CXCL12. This chemokine is constitutively expressed in bone marrow stromal cells as well as various organs including brain, heart, lung, kidney, thymus, spleen, and liver and has been proposed to play a role in immune surveillance, B-cell progenitor proliferation, B-cell migration and chemotaxis of monocytes, resting T lymphocytes and primitive haematopoietic cells (Nagasawa *et al*, 1994; Jo *et al*, 2000).

In the present study, which is the first to evaluate CXCL12/CXCR4 expression and its potential function in RCC, CXCL12α expression was decreased in kidney cancer compared to normal kidney tissue. None of the four tested RCC cell lines expressed significant amounts of CXCL12α mRNA. This may reflect their cancerous nature basing on the observation that CXCL12α is not expressed in premalignant colonic adenocarcinoma and reduced in the majority of hepatoma and digestive tract carcinomas when compared with adjacent non-cancerous tissue (Shibuta *et al*, 1997; Begum *et al*, 1999). It seems that CXCL12 counteracts carcinogenesis acting as a tumour-suppressor gene like molecule (Shibuta *et al*, 1997; Jordan *et al*, 1999). This effect may also be mediated by the ability of CXCL12α to inhibit the neovascularisation required for tumour growth. Thus, inhibition of CXCL12α production by malignant cells may be advantageous for the growth of certain tumours (Moore *et al*, 1998) – a concept that has not been unopposed (Salcedo *et al*, 1999).

CXCR4 is constitutively expressed in a broad range of tissues, including lymphatic tissues, thymus, brain, spleen, stomach, and small intestine (Nagasawa *et al*, 1994). The functions of CXCR4 have not yet been fully understood, but it appears that it mediates a large variety of functions including leukocyte development and trafficking, HIV-infection, correct foetal vascularisation and CNS development and possibly cell division, tumourigenesis and growth (Tachibana *et al*, 1998; Zou *et al*, 1998; Gabuzda and Wang, 1999). Rempel *et al* (2000) observed a tumour grade-dependent surge of CXCR4 mRNA levels in glioblastoma multiforme and that this receptor is mainly expressed in regions of angiogenesis and degenerative, necrotic, and microcystic changes rather than in sections of rapid cell proliferation. These data were confirmed by others, moreover expression of anti-sense CXCR4 transcripts in glioblastoma cell lines caused neurite outgrowth and cellular differentiation; treatment with CXCR4 specific antibodies lead to inhibition of cell proliferation. Therefore, it was concluded that CXCR4 is required for the proliferation of human glioblastoma tumours (Sehgal *et al*, 1998a,b).

In a recent publication, CXCR4 was shown to be highly expressed in breast cancer cells, malignant breast tumours as well as metastases. CXCR4 signalling was associated with actin polymerisation, pseudopodia formation, and subsequently chemotactic and invasive response. On the other hand CXCL12 exhibited peak levels of expression in organs representing the first destinations of breast cancer metastasis, i.e. lymph nodes, lung, liver and bone marrow. Neutralising the CXCL12-CXCR4 interaction significantly impaired metastasis to regional lymph nodes and lung in mice. Therefore, it appears that the final distribution of metastases in breast cancer reflects the relative abundance of CXCL12 in the different organs (Muller *et al*, 2001). As the pattern of metastasis between breast and kidney cancer are comparable at least to some extent, similar mechanism of CXCR4/CXCL12 in tumour spread might also be active.

In the present study, we found an upregulated expression of CXCR4 mRNA in kidney cancer samples compared to adjacent normal tissue. Functional receptors were found on the cell surface of A-498 RCC cells. These findings are in accordance with previous reports dealing with epithelial and neuronal malignancies and indicate that CXCR4 is overexpressed in rather dedifferentiated cells and/or regions of angiogenesis, degeneration and necrosis. All those cell forms can frequently be found in kidney cancer tissue. However, as A-498 but not CAKI-1, CAKI-2 or HA-7 cancer cells exhibited high CXCR4 mRNA expression levels, conditions like dedifferentiation, proliferation and angiogenesis on one hand or degeneration and necrosis on the other are certainly not solely and exclusively responsible for the regulation of CXCR4 expression.

To further elucidate molecular changes induced by CXCR4-mediated signalling, we employed cDNA expression arrays and compared CXCL12α stimulated A-498 cells (for 6 and 18 h, respectively) with unstimulated cells. After 6 h of stimulation, 31 genes involved in various cellular processes were found to be specifically up- or downregulated.

Concerning common signal transduction pathways also involved in CXCR4 signalling, several molecules were upregulated. A most interesting molecule in this group was CD82 (also termed kangai 1, suppressor of tumourigenicity 6, KAI1), as it was the only molecule being upregulated after 6 h (factor, 2.3) and 18 h (factor, 2.2) of CXCL12α stimulation, respectively. The transmembrane protein CD82 is directly associated with the epidermal growth factor receptor (EGFR) and other tyrosine kinase receptors and accelerates desensitisation of tyrosine receptor induced signalling correlated with an increased rate of receptor endocytosis (Odintsova *et al*, 2000). This attenuation of EGFR signalling by CD82 explains for its recently identified role as metastasis suppressor gene for prostate and pancreatic cancer (Dong *et al*, 1995; Guo *et al*, 1996). In addition, CD82 was described to be an activation/differentiation marker of mononuclear cells (Lebel-Binay *et al*, 1995). Other specifically regulated signal transduction molecules are given in Table 2.

The early growth response gene EGR1 was the only transcription factor being constantly downregulated by rhCXCL12α (6 h factor, −3.9; 18 h factor, −2.1). EGR1 was shown to be overexpressed in prostate cancer and to be involved in the regulation of different steps involved in the initiation and progression of prostate cancer, including mitogenesis, invasiveness, angiogenesis, and metastasis (Svaren *et al*, 2000).

Two members of the AP-1 transcription factor family, i.e. Fra-1 (factor, +4.5) and c-Jun (factor, +2.1), were significantly upregulated after 6 h. After 18 h of rhCXCL12α stimulation, c-Jun levels were significantly downregulated (factor, −1.9; data not shown). These altered c-Jun – Fra-1 ratios are interesting as Fra-1 protein heterodimerises with different Jun proteins to form stable AP-1 complexes with low transactivation potential. Thus, Fra-1 represses AP-1 activity, which is known to be an important mediator of tumour promoter action (Yoshioka *et al*, 1995).

Several other transcription factors, i.e. fuse binding protein (FBP)-2, NF-κB p100 subunit and its feedback inhibitor I-κBα as well as interferon regulatory factor (IRF)-1, showed only a transient upregulation after 6 h but not 18 h of treatment. IRF-1 functions as a regulator of the interferon system and key transcription factor in the regulation of cell cycle and apoptosis. It has been demonstrated to have antiproliferative and tumour suppressive functions, particularly through its transcriptional activation of IFN-α and IFN-β (Vaughan *et al*, 1997; Um *et al*, 2000).

rhCXCL12α also has an interesting but transient (i.e. 6 h) effect on cell cycle regulatory molecules. The G1-phase cyclin-D as well as the blockers of E2F-mediated transcription of S phase genes, the aryl hydrocarbon receptor (AHR) as well as the retinoblastoma-binding protein p48 (RbAp48), were significantly upregulated. On the other hand, cylcin-G1 levels were lower in rhCXCL12α treated cells. Cyclin-G1 expression has been shown to be tightly regulated throughout the cell cycle in normal cells, peaking at the S and G2/M phases of the cell cycle with lower levels in G1 (Reimer *et al*, 1999). Thus, CXCR4 signalling seems to have a temporary cell cycle inhibiting effect on A-498 cells preventing transition from G1 to S phase.

This cell cycle arresting effect is accompanied by an again transient upregulation of different pro-apoptotic molecules, i.e. procaspases-8, -10 and -6, caspase and RIP adapter with death domain (CRADD). While the first two caspases are initiator caspases, activated through death receptors like Fas or TNFR-1 and other stimuli, caspase-6 is an executioner caspase activated by the apoptotic cascade. This increased expression of pro-apoptotic molecules is paralleled by the neutralisation of the anti-apoptotic signalling through TNFR induced NF-κB by upregulated I-κBα (Mustapha *et al*, 2000).

In summary, it appears that in the case of CXCR4 expressing RCC, the CXCL12-CXCR4 pathway might be an interesting therapeutic target. The temporary and synchronous upregulation of a metastasis inhibiting proteins (e.g. CD82), of proapoptotic molecules (e.g. procaspases-8, -10 and -6, CRADD, I-κBα) and of negative cell cycle regulators (e.g. cyclin-D, AHR, IRF-1, RbAp48) is paralleled by a downregulation of molecules involved in cell cycle progression and metastasis (e.g. EGR1, cyclin-G1). Therefore, CXCL12 seems to be a promising molecule useful for the presensibilisation of tumour cells in a combination-therapy of RCC. However, further investigations are needed to determine a potential biological and clinical impact on metastasis and tumour progression.
